# The mitochondrial genome of the western bean cutworm, *Striacosta albicosta* (Lepidoptera: Noctuidae)

**DOI:** 10.1080/23802359.2016.1192499

**Published:** 2016-07-10

**Authors:** Brad S. Coates, Craig A. Abel

**Affiliations:** a United States Department of Agriculture, Agricultural Research Service, Corn Insects & Crop Genetics Research Unit, Ames, IA, USA;; b Department of Entomology, Iowa State University, Ames, IA, USA

**Keywords:** Invasive species, next generation sequencing, pest insect

## Abstract

The complete 15,553 bp mitochondrial genome of the western bean cutworm, *Stricosta albicosta*, (Lepidoptera: Noctuidae) was assembled from paired end Illumina HiSeq2500 read data. Annotation showed 13 predicted protein coding genes (PCGs), 22 tRNAs and 2 rRNAs have an order and orientation typical of insect mitochondrial genomes, and the derived rearrangement of tRNA-Met, -Ile and -Gln upstream of *nad*2 as in other Lepidoptera. A 79.3% A + T content resulted in a bias for codons with A or T in the 3rd position, and prevalence of synonymous substitutions suggest the effects of purifying selection on the mitochondrial genome sequence. Two microsatellite repeat motifs, (CA)_10_(AT)_19_ and (AT)_12_, are respectively located in intergenic spaces between tRNA-Glu and -Phe and tRNA-Leu and 16S rRNA. Mitochondrial phylogenomics was able to resolve sub-families within the Noctuidae, and suggest analogous analyses may be applicable across other lepidopteran Families.

The western bean cutworm, *Striacosta albicosta*, is a pest insect that primarily feeds on dry beans and corn crops grown in its native range in the high plains of North America. A recent eastward range expansion of this insect that started in the 1990s has resulted in periodic significant damage being caused to corn crops throughout the Midwest region of the United States (Michel et al. 2010). Damage to corn plants occurs primarily by larval feeding on grain producing reproductive tissues, and cannot be completely controlled by commercial transgenic corn hybrids that express the *Bacillus thuringiensis* (Bt) toxins Cry1Ab or Cry1F. This was demonstrated through high levels of resistance among *S*. *albicosta* in a laboratory colony (Dyer et al. 2013), and decreased susceptibility in field populations over the past decade (Ostrem et al. 2016). Despite the crop damage caused by this pest insect, associated genetic data remains limited.

We collected *S*. *albicosta* moths near Brule, Nebraska (Latitude 41.09, Longitude −101.89). A Next Generation Sequencing (NGS) approach (Coates 2014) resulted in a 15,553 bp complete assembled *S. albicosta* mitogenome, which has a high A + T nucleotide content (A = 40.1%; T = 39.2%; C = 7.9%; G = 12.8%) and is typical for most insects. The DOGMA annotation pipeline (Wyman et al. 2004) predicted13 predicted protein coding genes (PCGs), 22 tRNAs and 2 rRNAs (GenBank accession KM488268.1). Typical of Lepidoptera mitogenomes, tRNA-Met was translocated upstream of tRNA-Ile and just after the A + T-rich control region, which differs from the ancestral order among Insecta (Boore 1999) yet conserved among species of Lepidoptera (Coates et al. 2005). Similarly, a conserved alternate CGA (Arg) initiation codon was predicted for *cox*I and has also been described in the mitochondrial genomes of several Lepidoptera. Tandem repeat finder (Benson 1999) identified (CA)_10_(TA)_18_ and (AT)_12_ intergenic microsatellite regions, respectively, between tRNA-Glu and -Phe, and tRNA-Leu1 and *rrnL*.

Phylogenetic analysis of mitogenome sequences from the Family Noctuidae (Order Lepidoptera) confirmed placement of *S. albicoasta* within the sub-family Noctuinae (cutworms and dart moths) based on clustering with *Agrotis* sp. (Figure 1). In brief, the tree was constructed from an alignment of 3751 concatenated amino acid residues for 13 PCGs among mitogenomes from the Family Noctuidae (available in GenBank April 2016). After complete deletion of gapped sequences 3,675 characters were used for analysis. The subsequent Maximum Likelihood (ML) tree was constructed using the General Reversible Mitochondrial + Frequency + Gamma model of sequence evolution with pairwise distances estimated under the Jones, Taylor and Thornton (JTT) model of amino acid substitution (Jones et al. 1992). Node support was provided by 1,000 bootstrap pseudoreplications, and *Ostrinia nubilalis* (Lepidoptera: Crambidae) was the outgroup. Compared to prior analyses that placed subfamilies Heliothinae, Noctuinae, Hadenae and Amphipyrinae into a single polytomy (Speidel et al. 1996; Fang et al. 2000), our mitogenome phylogeny delineates these specific clades. Furthermore, our results suggest that full mitochondrial phylogenomic approach may be capable of resolving the relationships among species of Lepidoptera within the Family Noctuidae, and possibly applicable for analogous phylogenetic studies.

**Figure 1.**
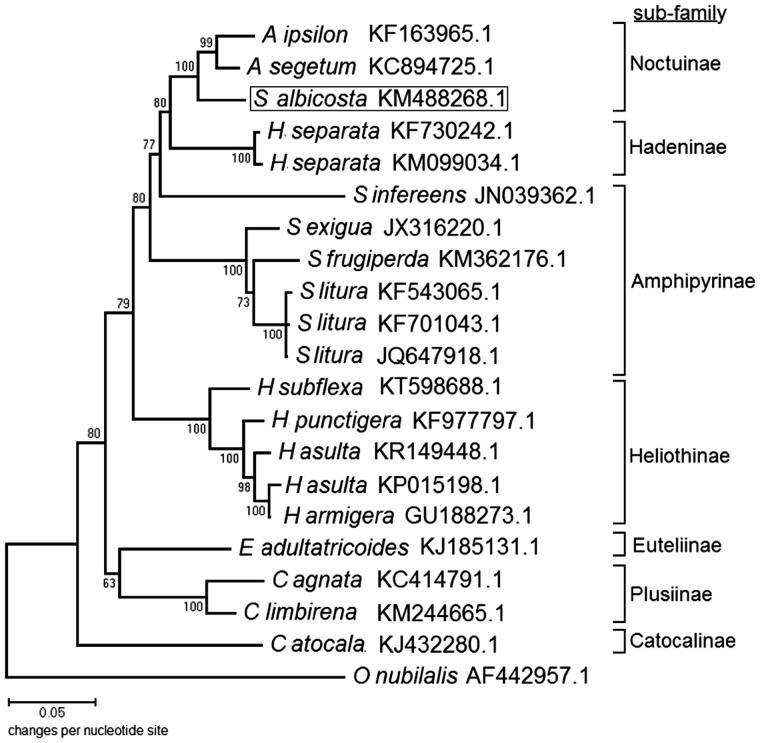
Maximum Likelihood based phylogenetic analysis of concatenated sequences derived from 13 PCGs in 20 full mitogenomes in the lepidopteran Family Noctuidae distributed across 7 sub-families. Number at each node is reported as the proportion of 1,000 bootstrap pseudoreplicates supporting each. Position of *S. albicoasta* is encoded in a box. Log Likelihood = −25962.89; gamma parameter = 0.2992.
